# Hybernation Considered in Its Phenomena Throughout the Animal Kingdom

**Published:** 1847-10

**Authors:** 


### Art. X.

*Der Winterschlaf nach seinen Erscheinungen im Thierreich dargestellt von* Dr. H. C. L. Barkow, ordentlichen Professor der Medicin und Director des Anatomie-Instituts an der königlichen Universität zu Breslau. Mit 4 Steintafeln.—*Berlin,* 1846.

*Hybernation considered in its Phenomena throughout the Animal Kingdom.* By Dr. H. C. L. Barkow, Ordinary Professor of Medicine and Director of the Anatomical Establishment in the Royal University of Breslau. With Four Lithographic Plates.—*Berlin,* 1846. 8vo, pp. 525.

This work contains a copious account of the phenomena of hybernation as it occurs throughout the animal kingdom, derived partly from the researches of preceding authors on the subject, but principally from the author's own observations, which have been very numerous, especially on the hybernation of snails, toads, hedgehogs, and marmots. Indeed, it is the number of his observations and the details he enters into in recording them, that has lengthened out the book to so many pages.

We cannot afford room for any elaborate analysis of the work, but must content ourselves with laying before the reader the following notices regarding the circumstances in the history of hybernation, most interesting in a physiological point of view.

There is no example of hybernation among birds, but among all the other classes of vertebrata and among the invertebrata there are examples. The duration of winter-sleep does not appear to be in any way connected with the class to which the animal belongs, for in animals of the same class it may be of long as well as of short duration, continued or interrupted. And, though in animals of the same species it is the same, there is probably some difference, according to age; seeing, for example, that Barkow found the winter sleep of younger individuals among hedgehogs in general shorter than that of older individuals.

The temperature of hybernating mammiferous animals, whilst in the waking state, is known not to be less than that of other mammifera, but, in common everyday sleep, Dr. Barkow says, that their temperature has a tendency to fall. During their winter-sleep the temperature exceeds little, or not at all, that of the atmosphere. The temperature is in the inverse ratio of the deepness of the sleep, being higher in the less degrees, and lower in the higher degrees. When cold weather is protracted the animals awake, and, if the cold be not severe, remain so; but, if the cold is severe, Dr. Barkow found that they became lethargic, stiff, and cold, and that they at last died.

*Respiration* during winter sleep is very slow, or may be wholly suspended. In the latter case *circulation*, however, still goes on, the action of the heart, though much diminished, not entirely stopping.

The following is the result of Dr. Barkow's observations on the blood of the hedgehog during winter sleep: Coagulation takes place slowly, as does also the separation of the clot and serum, and putrefaction is longer of taking place. In these respects the blood of the arteries (which is of a dark colour), is more remarkable than that of the veins; and the blood of old animals is more remarkable than that of young, arterial being compared with arterial blood and venous with venous; but the opposite is the case, if we compare the arterial blood of young animals with the venous blood of old.

Our author's observations agree with those of Dr. M. Hall, as to irritability being retained longer by the muscles in animals killed during winter sleep than when in the waking state; and as to the excitability of the parts of the nervous system engaged in the production of the reflex movements remaining.

Some animals, whose winter sleep is incomplete, such as the badger, the hamster perhaps, &c., do not entirely cease taking food, but by far the greater number of winter sleepers take no nourishment. When awakened by exposure to a warm temperature, Dr. Barkow found that they have in general a disinclination to eat, and if they do eat, the quantity taken is small, and in some it is imperfectly digested. Dr. Barkow also found that when the awakened state was kept up the animal died.

*Causes and nature of winter sleep.* Winter sleep occurs in animals with the most different forms and degrees of development of the nervous system, and with the most different forms of the vascular system. Nor in the blood is there any peculiarity which can be considered as a cause of winter sleep. Barkow thinks that imperfect respiration is the principal condition of winter sleep. But it may be asked, what is the cause of this imperfect respiration but a diminution or suspension of the respiratory movements, occasioned by a diminution or suspension of the nervous influence to the respiratory muscles; this diminution or suspension of nervous influence to the respiratory muscles being the result of some change of state of the nervous centre induced or promoted by diminution of external temperature?

Dr. Barkow found that, during winter sleep, young toads and young hedgehogs do not grow, and that regeneration of lost parts in snails is arrested. Retardation, he says, is the general character of the organic as well as of the animal functions. The fully developed animal passes slowly into winter sleep, and comes forth from it in a state of rejuvenescence. The tame marmot is found to have forgot its education and to have become wild again.

Winter sleep, Dr. Barkow affirms, is not of the nature of asphyxia. Hedgehogs in a state of winter sleep, if kept a long time under water, become asphyxiated, but the animal does not pass directly from the state of winter sleep to that of asphyxia, as from a lower to a higher degree of one and the same state, but first awakes, though imperfectly, and endeavours to escape. Again, the asphyxiated animal does not, on the return of life, pass through the state of winter sleep into the waking state, but directly into the latter, and only again falls into the winter sleep from the waking state. Nor has winter sleep any analogy, Barkow says, to the embryonic state, seeing that in this, development is most active, whilst in that, the activity of development is arrested.

Winter sleep differs essentially from common sleep. The most important differences between the two refer to digestion, growth, nutrition, reproduction, and formation of fat, almost all secretions, circulation, respiration, and evolution of heat. In common sleep, chymification goes on unchanged. Only at the beginning of incomplete winter sleep, or on interruption of it, does digestion go on. In perfect winter sleep the stomach is empty. If animals, with their stomachs full, are surprised by the sudden occurrence of cold, and put into winter sleep too soon, chymification stops. The common sleep favours nourishment, growth, and reproduction; winter sleep stops these processes. The common sleep of winter-sleepers powerfully promotes the formation of fat; during their winter sleep the fat is exhausted.

---

